# Genome announcement of *Steinernema khuongi* and its associated symbiont from Florida

**DOI:** 10.1093/g3journal/jkab053

**Published:** 2021-02-24

**Authors:** Anil Baniya, Peter DiGennaro

**Affiliations:** Department of Entomology and Nematology, University of Florida, Gainesville, FL 32611, USA

**Keywords:** genome, nematode, endosymbiont, entomopathogenic

## Abstract

Citrus root weevil (*Diaprepes abbreviates*) causes significant yield loss in citrus, especially in Florida. A promising source of control for this pest is biological control agents, namely, native entomopathogenic nematodes (EPNs) within the genus *Steinernema*. Two species of endemic EPN in Florida are *S. diaparepesi*, abundant within the central ridge, and *S. khuongi*, dominating the flatwood regions of the state. These citrus-growing regions differ significantly in their soil habitats, which impacts the potential success of biological control measures. Although the genome sequence of *S. diaprepesi* is currently available, the genome sequence of *S. khuongi* and identity of the symbiotic bacteria is still unknown. Understanding the genomic differences between these two nematodes and their favored habitats can inform successful biological control practices. Here, MiSeq libraries were used to simultaneously sequence and assemble the draft genome of *S. khuongi* and its associated symbionts. The final draft genome for *S. khuongi* has 8,794 contigs with a total length of ∼82 Mb, a largest contig of 428,226 bp, and N50 of 46 kb; its BUSCO scores indicate that it is > 86% complete. An associated bacterial genome was assembled with a total length of ∼3.5 Mb, a largest contig at 116,532 bp, and N50 of 17,487 bp. The bacterial genome encoded 3,721 genes, similar to other *Xenorhabdus* genomes. Comparative genomics identified the symbiotic bacteria of *S. khuongi* as *Xenorhabdus poinarii*. These new draft genomes of a host and symbiont can be used as a valuable tool for comparative genomics with other EPNs and its symbionts to understand host range and habitat suitability.

## Introduction

Entomopathogenic nematodes (EPNs) are a group of nematodes that are cosmopolitan in distribution and cause disease in insects. Commercially used as biological control agents for soil insects in the field and the greenhouse systems (Georgis *et al.* 2006; Denno *et al.* 2008; Lacey and Georgis 2012; Nicolopoulou-Stamati *et al.* 2016), these nematodes are broadly classified into two families, Steinemernatidae and Heterorhabditidae (Kaya and Gaugler 1993). Each family is symbiotically associated with a specific genus of entomopathogenic bacteria *Xenorhabdus* and *Photorhabdus*, for Steinemernatidae and Heterorhabditidae, respectively (Adams *et al.* 2006). This close and specific association between EPN and bacteria form an insecticidal complex that is effective against a wide range of insect hosts (Stock and Blair 2008).

Citrus is one of the major crops of Florida, totaling over 56% of the total citrus produced in the United States (Cowell *et al.* 2018). Among different pests of citrus, the citrus root weevil, *Diaprepes abbreviatus* (Linnaeus) is one of the most damaging pests (Nigg *et al.* 2001; Campos-Herrera *et al.* 2019). This insect is polyphagous and can damage other crops and ornamental plants. It was first detected in Florida during the mid-1960s (Beavers and Selhime 1975) and is spreading to other states including Southern Texas and Southern California (Lapointe 2008). In Florida, over 100,000 acres of citrus are infested causing 72 million dollars in losses (Stuart *et al.* 2008). Citrus root weevil can result in the rapid decline of citrus trees and overwhelm the entire groove within a short time. The severity of the infection and increasing infestation of this pest has prompted the use of diverse management strategies including chemical and biological control. Within known biological control strategies, EPNs are one of the most well-studied and widely used for citrus root weevil in Florida (Campos-Herrera *et al.* 2013; Duncan *et al.* 2013). Different species of commercially available nematodes within the genus *Steinernema* and *Heterorhabditis* have been tested in laboratory and field settings (McCoy *et al.* 2000; Shapiro and McCoy 2000; Duncan *et al.* 2013). Originally, *Steinernema carpocapsae* and *Heterorhabditis bacteriophora* were used but then replaced by *S. riobrave* and *H. indica* due to a shown greater efficacy against *D. abbreviatus*, based on insects mortality (Stuart *et al.* 2008). However, field efficacy of nonnative EPN to control the weevil was variable and some applications failed to control insect populations. Major factors responsible for the variability of the applied treatments are a regional variation of soil habitat (Duncan *et al.* 2001; McCoy *et al.* 2002) and diverse community structures of native EPN within different ecological regions (Duncan *et al.* 2003, 2007). In Florida, citrus is grown in two distinct climates, flatwood and central ridge. The infection rates of native EPN to citrus root weevil larvae varied between these ecoregions with higher population diversity and lower insect damage among central ridges and vice versa among the flatwood region (Duncan *et al.* 2003; Futch *et al.* 2005). Within the different species of the native EPN, *S. diaprepesi* was isolated from the larvae of the citrus root weevil and was found to be highly effective in controlling the pest. However, the population of this nematode is more frequent within citrus orchards in the central ridge (Nguyen and Duncan 2002; El-Borai *et al.* 2007b; El-Borai *et al.* 2007a). In 2009 a novel population of a *Steinernema* species was recovered during the field study along the flatwood region of Florida (El-Borai *et al.* 2009). Initially, it was coined as “SxArc” as s member of *S. glaseri.* The occurrence of this species was frequent in the subsequent survey of nematodes (Nguyen and Buss 2011; Campos-Herrera *et al.* 2013; Campos-Herrera *et al.* 2016). Later it was identified as a new species *S. khuongi* based on morphological differences and DNA sequence analyses (Stock *et al.* 2019).

Despite the potential as a biological control agent, the lack of efficacy in the field has hindered the wide adaptation of EPN. Some effort has been made through artificial selection of desired traits such as increased tolerance to environmental conditions, desiccation, and ultraviolet light, as well as improved host-seeking ability, virulence, and resistance to nematicides. Most of these traits are unstable, have reduced overall fitness, or result in inbreeding depression resulting in poor efficacy (Burnell 2002; Glazer 2015; Lu *et al.* 2016). Native nematodes usually provide better insect control compared to commercially available nematodes as they are better adapted in the local environmental condition (Gaugler 1988; Gaugler *et al.* 1997; Duncan *et al.* 2003; Hiltpold 2015). Crucial traits determining the efficacy of EPN as a biological control agent are infectivity, persistence, and storage stability. Identification of new species, availability of genomic sequences, and comparative genomics can help to address which genetic features contribute to the virulence and adaptability of one species to certain environmental conditions. This study aimed to simultaneously sequence the nematode *S. khuongi* and its bacterial symbiont native to the Florida flatwood region. Our goal was to identify the bacterial associate and characterize different genomic features of both nematode and bacteria to aim in informing novel EPN biological control agent development.

## Materials and methods

### Nematode culture

EPN infective juveniles (IJs) of *S. khuongi* “webber” strain were originally isolated from Florida citrus groves, and then cultivated in *Galleria mellonella* (waxworm) larvae at Citrus Research and Education Center (CREC), Lake Alfred, FL. Some strains were received from CREC and reinoculated into the wax worm, after emerging IJs were collected using modified White traps (Kaya and Stock 1997) at the Department of Entomology and Nematology, Gainesville, FL. Nematodes were stored in a tissue culture flask for subsequent identification, genomic DNA extraction, and to maintain the culture (Stock and Goodrich-Blair 2012).

### DNA isolation

Approximately 10,000 IJs were washed 10 times with a 0.8% NaCl solution. Surface sterilization of nematode was done by using 4 mM Hyamine 1622 solution (Sigma-Aldrich, USA) for 30 minutes and again washed with 0.8% NaCl for 3 times (Lu *et al.* 2017). The sterilized nematode were flash-frozen and thawed immediately twice for DNA extraction. High molecular weight genomic DNA was extracted using a phenol-chloroform method (Donn *et al.* 2008). The DNA pellet was further resuspended in 100 μl Tris-EDTA buffer. University of Florida’s campus-wide Interdisciplinary Center for Biotechnology Research (ICBR) NextGen DNA Sequencing Core Facility (Gainesville, FL) performed library preparation and sequencing using MiSeq Illumina sequencing platform with 2X300v3 format.

### Genome assembly

**Figure 1:**
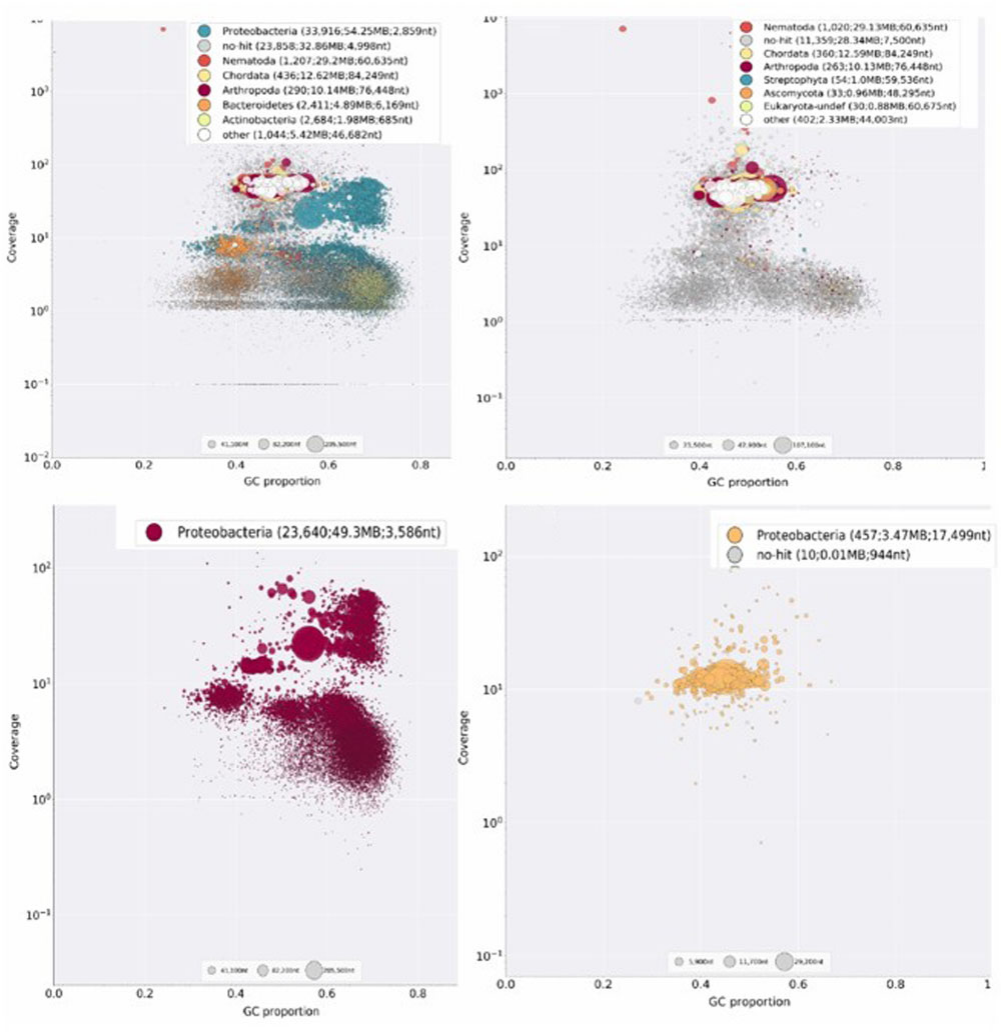
Blobplot (Taxon annotated GC-coverage scatter plot) of the contigs from the genome assembly. Each contig is plotted respective of their GC content and the depth of coverage. Each dot plot represents the contigs for the BLAST annotation with significant matches that are colored by putative taxon of origin. (A) Contigs from the preliminary assembly before removing the contaminants and the endosymbionts. (B) All contigs from the final assembly of the nematode draft genome. (C) All Putative proteobacterial contigs from preliminary assembly. (D) Contigs of the reassembled endosymbionts genome.

A total of ∼24 million 300-nt reads were generated from the sequencing. The sequence quality of the raw reads was analyzed using FastQC (Andrews 2010). Quality trimming, read filtering, and removing adapter contamination were performed using Trimmomatic/0.36 (Bolger *et al.* 2014). Clean reads were subjected to *de novo* assembly using the SPAdes/3.13.0 assembler (Bankevich *et al.* 2012) with Kmer size of 21, 33, 55, 77, 99, and 127. Assembly obtained from kmer 127 was used for downstream evaluation based on the best N50 value. The preliminary genome assembly of the nematode was likely contaminated with the symbiotic bacteria and other contaminants. To remove the possible contaminant from the assembly all contigs were queried against the NCBI nucleotide database using megablast (with E-value cutoff < 1e-05) and taxonomy was assigned to each contig (McGinnis and Madden 2004). Each contig was mapped and indexed to the raw read using Bowtie2 (Langmead and Salzberg 2012). Diagnostic visualization of each contig based on GC%, coverage, and taxonomic annotation was done using Blobtools v1.0 (Kumar and Blaxter 2011; Laetsch and Blaxter 2017). Based on the taxonomic annotation of each contig in Blobplot (Figure 1) each contigs sets belonging to nematode and proteobacteria were separated. After removing all the contaminants from the nematode set of contigs, the completeness of the genome was assessed using BUSCO V3.02 (Simão *et al.* 2015) and the nematode BUSCO profile (https://busco-archive.ezlab.org/v3/datasets/nematoda_odb9.tar.gz). Nematode BUSCO genes are expected to be the core genes and most probably present in every newly sequenced genome. A higher number of BUSCO genes in the assembly is indicative of completeness of the assembly. The quality of the assembly and the genome statistics were determined using Quast 2.3 (Gurevich *et al.* 2013).

### Reassembly of bacteria, annotation, and comparative genomics

**Figure 2:**
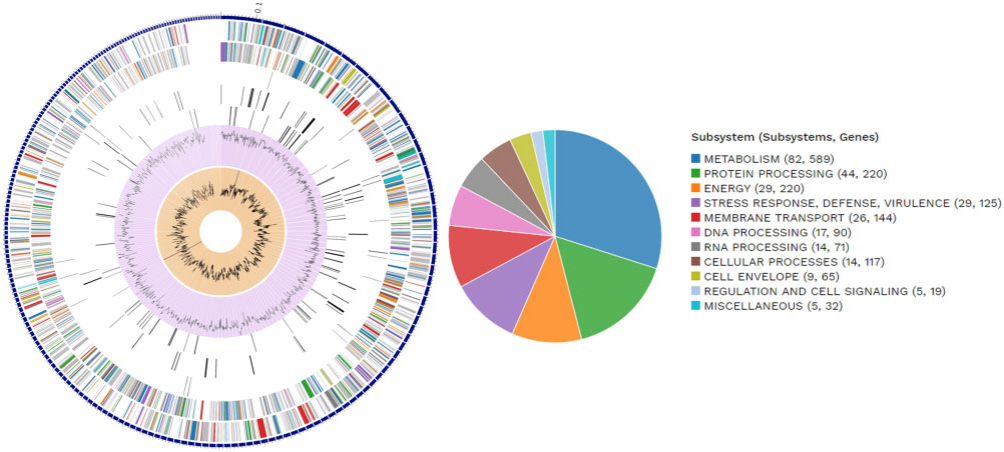
Graphical display of bacterial genome annotation. (A) Circular graphical display of the distribution of the bacterial genome annotations. The outermost region is the contigs, followed by CDS on the forward strand, CDS on the reverse strand, RNA genes, CDS with homology to known antimicrobial resistance genes, CDS with homology to known virulence factors, GC content and GC skew, respectively. The colors of the CDS on the forward and reverse strand indicate the subsystem genes. The circular display has been limited to the 215 longest contigs of the 468 contigs in the genome. (B) The subsystem annotation of the genome that implements a specific biological process.

**Figure 3:**
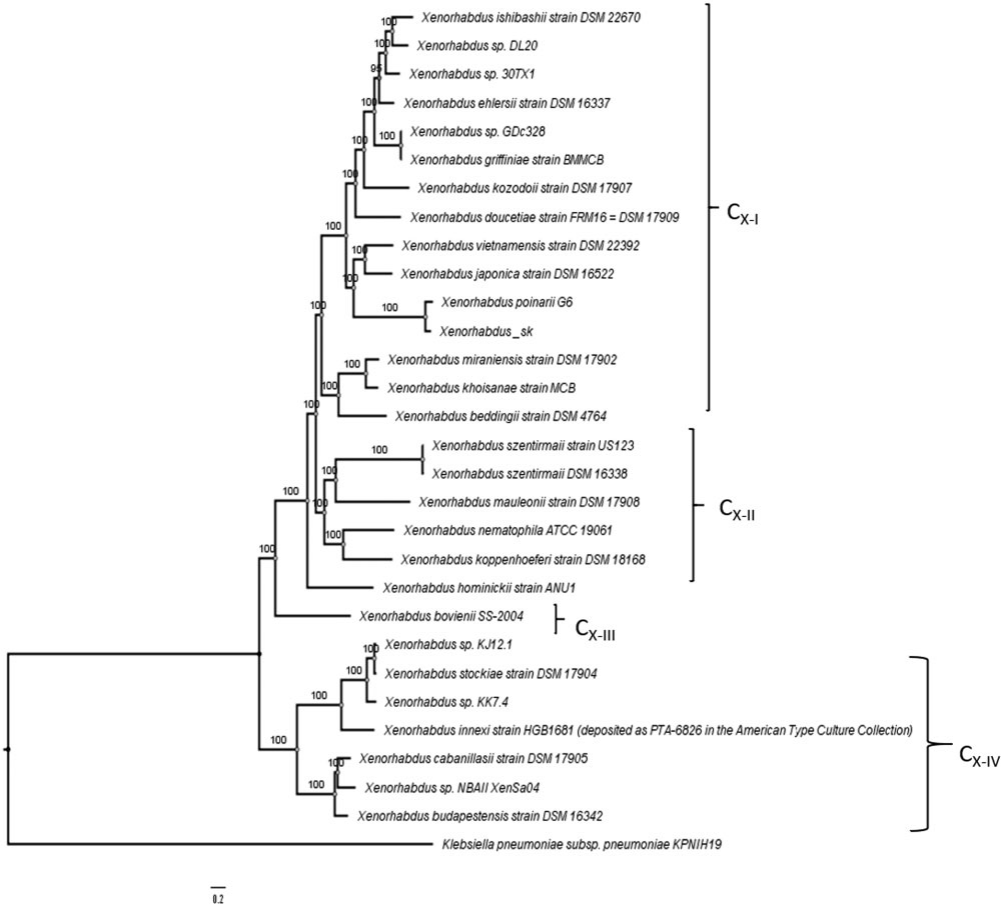
Phylogenetic tree of the selected species of *Xenorhabdus*. Phylogenetic tree based on a combined analysis of amino acid and nucleotide sequences of single-copy genes from each species. The tree is drawn by using Randomized Axelerated Maximum Likelihood (RAxML version 8) with 100 bootstrap replicates. Bootstrap value for each node is shown. The tree is drawn to scale. Clades are labeled according to Tailliez *et al.* (2010).

**Figure 4:**
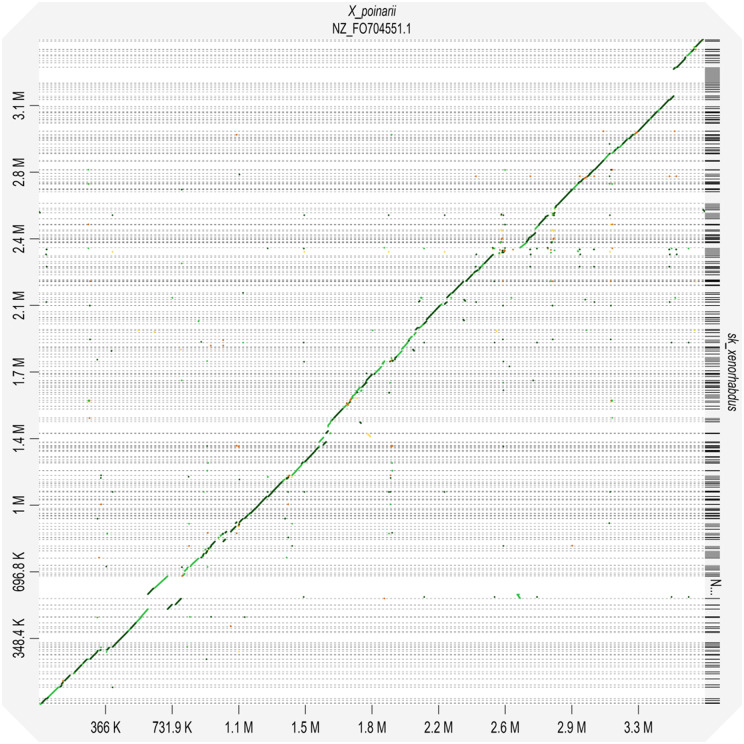
D-GENIES dot plots (using Minimap2 aligner) indicating collinearity of *Xenorhabdus* sp from *S. khuongi* with the *Xenorhabdus poinarii* str G6 from *S. glaseri*.

**Figure 5:**
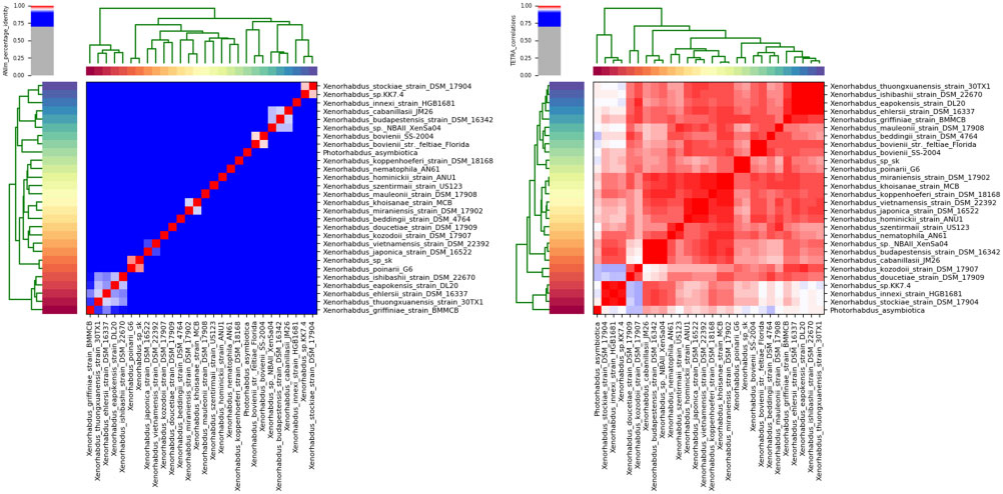
Heatmap of average nucleotide identity based on MUMmer (ANIm) based on whole genome alignment of 26 *Xenorhabdus* species *1 Photorhabdus asymbiotica* and *Xenorhabdus* sp from *S. khuongi*. The left heatmap shows ANIm percentage identity. Dendrogram shows similarity according to ANIm identity scores. The right heat map shows the Tetra nucleotide frequency correlation coefficient of the above-mentioned species genome alignment.

Each *Steinernema* species are symbiotically associated with only one specific species of the genus *Xenorhabdus* (Enterobacteriaceae, which belongs to the gamma subdivision of the Proteobacteria) (Stock and Blair 2008). To assemble the *Xenorhabdus* spp. of the *S. khuongi* First we retained all contigs classified as Proteobacteria in the preliminary nematode assembly. We then filtered all the contigs belonging to other proteobacterial species with a high level of identity. We extracted all the read mapped to the contigs to generate the *Xenorhabdus*-enriched raw sequence dataset. These putative *Xenorhabdus* raw reads were reassembled using Spades/3.13.0 assembler using optimized settings for bacterial genome assembly (Bankevich *et al.* 2012). The quality of the genome assembly was determined as described above. Annotation of the bacterial genome was done by using Pathosystems Resource Integration Center (PATRIC) RASTtk-enabled Genome Annotation Service (Brettin *et al.* 2015) and shown in Figure 2. Known homologs to antibiotic-resistance genes, drug targets, transporter, and virulence factor genes were identified using PATRIC by known homology of known sequences in the following databases: Comprehensive Antibiotic Resistance Database (CARD) (McArthur *et al.* 2013), DrugBank 4.0 (Law *et al.* 2014), Therapeutic Target Database (TTD) (Zhu *et al.* 2010), Transporter Classification Database (TCDB) (Saier *et al.* 2016), PATRIC_VF (Mao *et al.* 2015), Virulence Factor (VFDB) (Chen *et al.* 2016), and the National Database of Antibiotic-Resistant Organisms (NDARO). A k-mer-based AMR gene detection method was used to annotate antimicrobial resistance (AMR) genes, which utilizes PATRIC’s curated collection of representative AMR gene sequence variants (Wattam *et al.* 2017). To characterize the closest relative of the newly sequenced bacterial genome we generated the phylogenetic tree using a codon tree in PATRIC (Wattam *et al.* 2017). Genomes 28 previously sequenced *Xenorhabdus* species (Table 1) were used to generate the tree. The codon tree used cross-genus families (PGFams) as homology groups. 100 single-copy genes were selected from each genome. The alignment of each protein sequence was done using MUSCLE (Edgar 2004), and their corresponding nucleotide were aligned using the codonalign function of Biopython (Cock *et al.* 2009) within PATRIC (Davis *et al.* 2020). Concatenated trees of proteins and nucleotide were generated from 100 rounds of bootstrapping in RAxML analysis (Stamatakis *et al.* 2008; Stamatakis 2014). *Klebsiella pneumoniae* subsp*. pneumoniae KPNIH19* was used as an outgroup. The trees were visualized using FigTree software version 1.4.4 (http://tree.bio.ed.ac.uk/software/figtree/) and shown in Figure 3. We assessed the synteny of newly assembled bacteria and *X. poinarii* str G6 using a web-based program called D-GENIES (Cabanettes and Klopp 2018), and using minimap2 for alignment, shown in Figure 4. To confirm the identity of the bacteria, the genome sequence was compared with other previously reported genomes from other *Xenorhabdus* spp. using Pyani version 0.2.7 (Pritchard *et al.* 2016). Pyani is a Python 3 module that can calculate Average Nucleotide Identity (ANI) using MUMmer and blast. Pyani was also used to calculate tetra-nucleotide frequencies (TETRA) correlation indexes. In this study, 26 complete genomes of different species/strains of *Xenorhabus* and one species of *Photorhabdus* were analyzed (Figure 5).

**Table 1:** List of *Xenorhabdus* spp. with NCBI Taxon ID and PATRIC genome ID numbers considered for the phylogenetic studies

NCBI Taxon ID	PATRIC Genome ID	Genome Name
351659	351659.4	*Xenorhabdus koppenhoeferi strain DSM 18168*
626	626.21	*Xenorhabdus _sk*
351673	351673.4	*Xenorhabdus cabanillasii strain DSM 17905*
53341	53341.3	*Xenorhabdus japonica strain DSM 16522*
406818	406818.4	*Xenorhabdus bovienii SS-2004*
290111	290111.6	*Xenorhabdus ehlersii strain DSM 16337*
1034471	1034471.3	*Xenorhabdus ishibashii strain DSM 22670*
351671	351671.5	*Xenorhabdus doucetiae strain FRM16 = DSM 17909*
1354304	1354304.4	*Xenorhabdus poinarii G6*
1873484	1873484.3	*Xenorhabdus sp. 30TX1*
1873482	1873482.3	*Xenorhabdus sp. DL20*
351676	351676.4	*Xenorhabdus kozodoii strain DSM 17907*
1429873	1429873.3	*Xenorhabdus sp. NBAII XenSa04*
290110	290110.6	*Xenorhabdus budapestensis strain DSM 16342*
40578	40578.4	*Xenorhabdus beddingii strain DSM 4764*
351672	351672.4	*Xenorhabdus griffiniae strain BMMCB*
742178	742178.3	*Xenorhabdus sp. GDc328*
351656	351656.5	*Xenorhabdus vietnamensis strain DSM 22392*
406817	406817.27	*Xenorhabdus nematophila ATCC 19061*
351614	351614.4	*Xenorhabdus stockiae strain DSM 17904*
351679	351679.5	*Xenorhabdus hominickii strain ANU1*
351674	351674.5	*Xenorhabdus miraniensis strain DSM 17902*
290109	290109.7	*Xenorhabdus innexi strain HGB1681*
351675	351675.7	*Xenorhabdus mauleonii strain DSM 17908*
880157	880157.4	*Xenorhabdus khoisanae strain MCB*
1851572	1851572.3	*Xenorhabdus sp. KK7.4*
1851571	1851571.3	*Xenorhabdus sp. KJ12.1*
290112	290112.3	*Xenorhabdus szentirmaii strain US123*
1427518	1427518.5	*Xenorhabdus szentirmaii DSM 16338*
1128953	1128953.3	*Klebsiella pneumoniae subsp. pneumoniae KPNIH19*

## Results

### *de novo* assembly of nematode genome sequence

We sequenced the *S. khuongi* genome using the Miseq Platform generating ∼24 million raw reads, equivalent to 7 Gb (∼70x). The genomic DNA extraction for sequencing was done from the nematode without removing endosymbionts (proteobacteria), so it is inevitable that the preliminary assembly is contaminated with endosymbionts and other contamination. The preliminary assembly consists of 65,846 contigs with total length of 151,364,689 bp. The megablast of the preliminary contigs with E-value cutoff < 1e–05 and visualization of contigs in Blobplot divided the contigs into 8 different taxon groups (Nematoda ∼33%, Proteobacteria ∼13%, No distinct hit on database ∼22%, Chordata ∼12%, Arthropoda ∼10%, Bacteroidetes and Actinobacteria ∼1%, and Other ∼5%; Figure 1). All the contigs that were assigned for Proteobacteria, Bacteriodetes, and Actinobacteria were removed from the preliminary nematode contigs. The final assembly of the nematode was obtained with mean contigs coverage of ∼41x. The final draft genome has 8,794 contigs with a total length of 81,789,282 bp (∼82 Mb) with a largest contig of 428,226 bp. The average GC content of the genome is 47.88% with N50 of 46 kb. The BUSCO assessment of the draft genome against the Nematoda database indicated 86.6% (850) completeness. A total of 982 nematode genes were searched. Among those genes, 81.3% (798) are complete and single copy, 5.3% (52) complete and duplicated genes, 6.5% (64) fragmented genes, and 6.9% (68) missing genes. Comparisons of *Steinernema* genomes can be found in Table 2.

**Table 2:** Features of *Steinernema* spp. draft genomes

*Steinernema*	*Carpocapsae*	*Scapterisci*	*Feltiae*	*Glaseri*	*Monticolum*	*Feltiae (NW)*	*Diaprepesi*	*Khuongi*
Estimated genome size (Mb)	84.5	79.4	82.4	92.9	89.3	121.6	118	82
N50 (bp)	7,362,381	90,783	47,472	37,444	11,556	60,433	11,474	42,000
Number of scaffolds/ contigs	16	2,877	5,839	7,515	14,331	4,678	35,545	8,794
GC content (%)	45.7	47.98	46.99	47.63	42.01	NA	45.01	47.88
N content (Mb)	NA	0.76	2.76	3.37	4.34	0	0	0
N content (%)	0.54	0.96	3.36	3.64	4.87	0	0	
Maximum scaffold size (bp)	20,922,283	1,149,164	1,470,990	339,094	110,081	1,315,981	1,706,490	428,226
Predicted genes	91,957	31,378	33,459	34,143	36,007	32,304	NA	NA
Complete BUSCO	87%	84.5%	84.32%	59.4%	69.2%	87.27%	85%	86.6% (850)
Single-copy BUSCOs	NA	79.3%	80.55%	57.8%	65.4%	76.68%	79.60%	81.3% (798)
Duplicated BUSCOs	NA	5.2%	3.77%	1.6%	3.8%	10.59%	5.40%	5.3% (52)
Fragmented BUSCOs	7%	8.1%	8.15%	12.9%	12.5%	7.33%	7.10%	6.5% (64)
Missing BUSCOs	6%	7.4%	7.54%	27.7%	18.3%	5.40%	7.90%	6.9% (68)
**Reference**	Serra *et al.* (2019)	Dillman *et al.* (2015)				Fu *et al.* (2020)	Baniya *et al.* (2020)	This study

### Reassembly of bacteria, annotation, and comparative genomics

Approximately 2.5 million quality trimmed raw read was mapped to putative proteobacterial contigs from the first preliminary assembly of the genome. Raw read mapped to proteobacterial contigs contribute approximately 10% of the initial filtered read sets. Proteobacterial contigs belonging to the genus *Xenorhabdus* were filtered and its corresponding raw reads used for optimized bacterial genome reassembly resulted in 468 contigs with a total length of 3,484,021 bp (∼3.5 Mb). The longest contig of the genome is 116,532 bp long, a contig N50 of 17,487 bp, and an average GC content of 45%. Based on the annotation statistics and a comparison to other genomes in PATRIC within this same species, this genome appears to be of good quality. Annotation of the genome resulted in 3,721 protein-coding sequences (CDS), 29 transfer RNA (tRNA) genes, and 3 ribosomal RNA (rRNA) genes (Figure 2). The genome annotation included 940 are hypothetical proteins and the remaining 2781 proteins with functional assignments. Protein with the functional assignment included 906 proteins with Enzyme Commission (EC) numbers, 735 with Gene Ontology (GO) assignments, and 639 proteins that were mapped to KEGG pathways.

The taxonomic identification of symbiotic bacteria associated with *S. khuongi* is still unknown. To validate the identity and place the genome in the bacteria we constructed the phylogenetic tree applying a multigene approach using the whole genome sequences. Multigene phylogeny provides robust phylogeny compared to using single genes, and these resolved trees are essential to study the co-evolution of bacteria with their nematode hosts and the information can be used for the classification of strain and new isolate within the same genera (Tailliez *et al.* 2010). The phylogenetic tree built in the PATRIC server was similar to the phylogenetic tree derived from the distance analysis of four concatenated protein-coding sequences as described by Tailliez *et al.* 2010. Single-copy genes from 30 different strains/isolates of bacteria were used to construct the tree with the RAxML algorithm (Table 1). A total of 45,907 amino acids and 137,721 nucleotides were aligned to construct the trees (Figure 3). In the codon tree, bacterial sequences isolated from bacteria associated with *S. khuongi* (Xenorhabdus_sk) formed a cluster close to *Xenorhabdus poinarii* str. G6 (GeneBank Accession: FO704551.1) isolated from *S. glaseri* (Ogier *et al.* 2014) within clade C_X-I_ among the *Xenorhabdus* genus.

Endosymbiont *X. poinarii* is known to be associated with different species of EPNs for example *S. glaseri* (Akhurst 1986) *S. cubanum* (Fischer-Le Saux *et al.* 1999). These two already identified nematode species that harbor *X. poinarii* are phylogenetically related, and belong to the same clade within the *Steinernema* genera (Nadler *et al.* 2006; Stock *et al.* 2019). Phylogenetic classification of *S. khuongi* based on the large subunit region (28S) clustered *S. cubanum*, *S. glaseri*, and *S. khuongi* very close to each other into the same clade also known as “*glaseri*-group” of *Steinernema* spp. (Stock *et al.* 2019).

The genome assembly of *Xenorhabdus poinarii* str. G6 (GeneBank Accession: FO704551.1) was also annotated in PATRIC to compare the genome characteristics of the bacteria. The genomes of two strains have almost the same G + C content but some differences in the number of tRNA and rRNA genes. Sequencing, assembly, and annotation information of *Xenorhabdus* sp. sk and *Xenorhabdus poinarii* str. G6 is shown in Table 3.

**Table 3:** Assembly and annotation features of *Xenorhabdus* sp. sk and *Xenorhabdus poinarii* str. G6

Assembly information	*Xenorhabdus_ sp_sk*	*Xenorhabdus poinarii*
Assembly size (bp)	3,484,021	3,659,523
Number of contigs	468	1
G + C content (%)	44.7	44.55
Largest contig (bp)	116,532	3,659,523
N50 (bp)	17,487	3,659,523
L50	58	1
**Annotation information**		
CDS	3,721	3,706
rRNA genes	3	22
tRNA genes	29	77
Hypothetical proteins	940	1,010
Proteins with functional assignments	2,781	2,696
Proteins with EC number assignments	906	853
Proteins with GO assignments	735	700
Proteins with Pathway assignments	639	611
Proteins with PATRIC genus- specific family (PLfam) assignments	3,083	3,481
Proteins with PATRIC cross- genus family (PGfam) assignments	3,133	3,487
**Specialty genes/source**		
Antibiotic Resistance/CARD	14	14
Antibiotic Resistance/PATRIC	51	47
Drug Target/DrugBank	107	96
Drug Target/TTD	18	18
Transporter/TCDB	91	88
Virulence Factor/PATRIC_VF	38	35
Virulence Factor/VFDB	14	10
Virulence Factor/Victors	61	55

The D-GENIES genome-wide comparison of *Xenorhabdus* sk to the reference *X. poinarii* str G6 shows that these genomes are highly collinear at the genome level. The symbiotically associated bacteria of *S*. *khuongi* seem to be very close to *X*. *poinarii* (Figure 4).

Because of this close relationship, it may be difficult to speciate based on phenotypic characteristics. Speciation using the DNA-DNA hybridization (DDH) technique provides better insight into genomic interrelationship and offer a reliable answer to differentiating species (Rosselló-Mora and Amann 2001). The bioinformatics method that mirrors the DDH is the average nucleotide identity (ANI) between genomes (Richter and Rosselló-Móra 2009). Average nucleotide identity values (ANI %) of the whole-genome assembly were calculated between *S*. *khuongi* bacterial symbionts (*Xenorhabdus* sp. sk), 26 *Xenorhabus* genome assemblies available in GenBank, with *Photorhabdus asymbiotica* genome as an outgroup. An ANI calculation performed with MUMmer indicated that the genome *Xenorhabdus* sp. from *S. khuongi* had 97% sequence similarity with that of *X. poinarii* str. G6. Similarly, tetranucleotide correlation between these two species was 99.8%. Average nucleotide identity (ANIb) using BlastN at NCBI was also calculated whose result indicated that these two bacteria share 96.5% sequence identity. The average nucleotide identity (ANI) of 94 to 96% is generally used as the cutoff value to separate bacterial species based on their genome sequences (Konstantinidis and Tiedje 2005; Richter and Rosselló-Móra 2009). Not only does our newly sequenced genome share approximately 97% average nucleotide identity (ANIm and ANIb) with *X. poinarii* str G6, but it also has a higher tetranucleotide correlation coefficient of 99.8%. These results can be used as conclusive evidence to prove that the bacterial symbiont associated with *S. khuongi* is *X. poinarii* (Figure 5).

## Discussion

The symbiotic association of specific bacteria within the gut of EPNs makes the genome sequencing of only one partner difficult. Due to their close association, it is nearly impossible to get clean DNA of only one species. Working with the newly identified nematode species makes it more complex because of the unknown association of co-bionts. The use of a blob-plot allows each contig from the assembly are separated based on the taxonomy and GC-coverage and can easily discern the nematode, bacterial symbionts, and other contamination if any present, in the assembly (Figure 1). This approach proves to be a robust tool that reduces the cost of the sequencing and cuts the complexity of the host-symbionts system as a simple metagenomic project and provides better insight to differentiate the unique genomes captured during sequencing. This approach is also feasible for the system where lab culture and isolation of symbionts are not possible (Kumar and Blaxter 2011).

The nematode *S. khuongi* is endemic to the environmental condition of the Flatwood region of Florida Citrus orchards. This region has a devastating problem with the citrus weevil (Stuart *et al.* 2008)). The availability of the genome of the nematode and associated bacteria can serve as a valuable resource to analyze various genetic factors that enables this nematode to thrive in these specific environmental conditions, and that also further improve it as a more effective biological control agent. As we are reporting the draft genomes of the nematode and bacteria, we anticipate some genome variation between isolates sequenced in the future. Although methods like flow cytometry can accurately predict the size of the genome and check for heterozygosity, the genome reported here is similar to those within its genus. Because our genome has nearly 9,000 gaps, to improve the quality of the genome it is recommended to combine short reads like ours with additional sequencing of long-insert DNA libraries from other technologies. However, even in their current state, these genomes should be valuable tools for comparative and functional genomics. Rather than sequence members of a symbiotic association we sequenced, assembled, and analyzed the genome of both symbionts and host of the EPN in one sequencing. This method of experiment and analyzing the data saves the time and cost of sequencing, but also retrieves valuable information that is hard to recover using more conventional methods.

## Data availability

All genome sequencing data generated in this study for both nematode and bacteria are deposited at the NCBI database under the BioProject number PRJNA670677 for nematode and PRJNA670736 for bacteria.
